# Risk factors and mediation role of sleep quality for depression in cognitively frail older adults: a cross-sectional study

**DOI:** 10.3389/fnagi.2025.1541555

**Published:** 2025-05-30

**Authors:** Xiaoxing Lai, Yonghua Cai, Hongwei Zhu, Zhiyuan Zhang, Xinyue Zhang, Yang Li, Jiazhen Liu, Xiaopeng Huo

**Affiliations:** ^1^Department of Neurology, Peking Union Medical College Hospital, Beijing, China; ^2^Nursing Department, Peking Union Medical College Hospital, Beijing, China; ^3^Medicine Intensive Care Unit, Peking Union Medical College Hospital, Beijing, China

**Keywords:** cognitive frailty, depression, elderly, activities of daily living, sleep quality

## Abstract

**Objective:**

Aimed to investigate the risk factors associated with depression in community-dwelling older adults with cognitive frailty and to examine the mediating role of sleep quality in the relationship between activities of daily living (ADL) and depression.

**Methods:**

A cross-sectional study was conducted using convenience sampling, enrolling older adults with cognitive frailty from six communities in Beijing from July 2023 to December 2023. Cognitive frailty was assessed using the Montreal Cognitive Assessment (MoCA) alongside with the Fried Frailty Phenotype, while depressive symptoms were measured with the Geriatric Depression Scale (GDS-15). Multivariate logistic regression analysis was used to identify risk factors influencing depression, and mediation analysis was employed to explore the mediating effect of sleep quality on the relationship between ADL and depression.

**Results:**

Among the 529 elderly participants with cognitive frailty, 128 (24.2%) were found to exhibit depressive symptoms. Multivariate logistic regression identified ADL [Mild Dependence: OR = 176.729 (95% CI 32.427–963.172), *p* < 0.001; Moderate Dependence: OR = 51.769 (95% CI 12.541–213.697), *p* < 0.001], loneliness [OR = 13.821 (95% CI 6.095–31.338), *p* < 0.001], and sleep quality [Suspected Insomnia: OR = 7.310 (95% CI 2.316–23.074), *p* = 0.001] were significantly associated with depression. Sleep quality was found to mediate the relationship between ADL and depression, accounting for 2.82% of the total effect.

**Conclusion:**

Dependence in ADL, loneliness, and poor sleep quality are potential risk factors of depression for cognitive frailty in aging adults. Moreover, sleep quality was found to mediate the relationship between ADL dependence and depressive symptoms.

## Introduction

Cognitive frailty, prevalent among older adults, is characterized by the simultaneous presence of physical frailty and cognitive decline, distinct from conditions like Alzheimer’s disease (Sugimoto et al., 2022; Zhang et al., 2023). This phenomenon is a critical aspect of aging well, with reported prevalence rates exhibiting considerable variability, ranging from 1.0 to 22.0%, thereby constituting an increasing concern (Panza et al., 2018). A recent study estimated that approximately 9% of community-dwelling seniors are affected by cognitive frailty (Qiu et al., 2022). This condition is significantly associated with adverse health outcomes in older adults, including dementia, falls, disability, and a decline in functional ability, which subsequently contribute to increased healthcare expenditures and an elevated risk of mortality (Alkhodary et al., 2022; Rivan et al., 2021). Furthermore, the notable co-occurrence of cognitive frailty and depression among the elderly indicates a complex interaction between these two conditions that necessitates further exploration (Zou C. et al., 2023; Zou Z. et al., 2023).

Potential factors influencing depression in older adults with cognitive frailty are complex and involve an interplay of physical, cognitive, and psychosocial elements. Previous studies have shown that difficulties with activities of daily living (ADL) predict depressive symptoms, especially when there is a decline in self-care abilities, which correlates with a more severe degree of depression (Liu et al., 2023). Cognitive decline can also contribute to feelings of depression, as individuals may experience a sense of loss and diminished autonomy (Zacková et al., 2021). Additionally, social factors such as isolation, lack of social support, and loneliness are consistently associated with higher rates of depression in this population (Taylor et al., 2018).

Research suggests that sleep quality may play a mediating role in the relationship between ADL and depressive symptoms, particularly among older adults experiencing cognitive decline. Studies indicate that sleep disorders are common in older adults and tend to worsen with cognitive decline, creating a bidirectional relationship (Liu et al., 2022). This assertion is further corroborated by studies demonstrating a link between compromised ADL and an increased likelihood of sleep disturbances (Karam, 2022; Liu et al., 2023). Reduced mobility associated with cognitive frailty can lead to lethargy and diminished mental stimulation, potentially resulting in a decrease in both non-rapid eye movement and rapid eye movement sleep, as well as disruption of the sleep–wake cycle (Ren et al., 2024). Such disruptions can advance the sleep phase and increase the risk of circadian rhythm disorders, thereby contributing to depressive symptoms. Furthermore, studies conducted by Wu et al. (2023) and Boga and Saltan (2020) have identified significant associations between sleep quality, mental status, and daily living activities, with depression mediating some of these relationships. Additionally, Ren *et al*. found a chain mediation effect of ADL and depression on the relationship between sleep quality and health-related quality of life (Ren et al., 2024). Nevertheless, the intricate relationship between ADL, sleep, and depression emphasizes the necessity for a comprehensive understanding of how these factors interconnect, particularly in the context of cognitive frailty.

This study aims to investigate the factors associated with depression in cognitively frail older adults, and to examine the mediating role of sleep quality in the relationship between ADL and depressive symptoms.

## Methods

### Study design and study population

This cross-sectional study was conducted across six communities in Beijing between July and December 2023. The inclusion criteria were as follows: (1) Individuals aged 60 years or older; (2) Those who meet the cognitive frailty criteria, which consist of a Montreal Cognitive Assessment (MoCA) score below 26, with an additional point allotted for individuals with less than 12 years of education; a Fried Frailty Phenotype (FFP) (Martin and O'Halloran, 2020) score of 3 or higher; no diagnosis of dementia, and a Clinical Dementia Rating (CDR) score of 0.5; (3). Participants who provided informed consent, were voluntary, and were capable of normal communication and cooperation to complete the study. The exclusion criteria included: (1) Individuals with a clinical diagnosis of dementia; (2) Patients exhibiting severe organ function decline or in the terminal phase of an illness; (3) Those with significant hearing, vision, or language impairments, or other physical conditions that would hinder their ability to complete the survey assessment. Informed written consent was requested before permission to participate was granted. This study was conducted with approval from the Ethics Committee of Peking Union Medical College Hospital (ZS-2943).

### Data collection

Questionnaires were administered by trained researchers utilizing an online survey software platform known as Wenjuanxing (powered by www.wjx.cn), which automatically flagged missing responses to ensure data integrity. Questionnaires were considered invalid if they contained incomplete information, more than three unanswered questions, evident contradictions, unusually brief completion times, or patterned responses.

General demographic data were collected, including gender, age, socioeconomic status, education level, living arrangements, marital status, monthly household income, chronic health conditions, and family medical history of the participants.

The MoCA, created by Nasreddine and his colleagues, is an enhancement of the Mini-Mental State Examination (MMSE). It includes 30 items that assess eight cognitive domains. A score below 26 points indicates cognitive impairment (Nasreddine et al., 2005). The FFP, developed by Fried and colleagues, is grounded in the frailty cycle model and employs five key diagnostic criteria: unintentional weight loss, reduced grip strength, slower walking speed, decreased physical activity, and self-reported exhaustion. An elderly individual is classified as frail if they meet three or more of these criteria. Meeting one or two criteria suggests a pre-frailty state, while the absence of any criteria indicates non-frailty (Abizanda et al., 2016). The Athens Insomnia Scale (AIS) was utilized to assess participants’ sleep quality over the preceding month (Soldatos et al., 2020). The scoring criteria are as follows: a total score below 4 indicates the absence of sleep disturbances, a score between 4 and 6 suggests the possibility of insomnia, and a score above 6 confirms the presence of insomnia. Additionally, the Epworth Sleepiness Scale was employed for further evaluation, with scores ranging from 0 to 24 points. A total score exceeding 6 points indicates the presence of excessive sleepiness (Kendzerska et al., 2014). The Social Support Rating Scale (SSRS) was used in this survey. This 10-item scale evaluates four dimensions of social support: objective support, subjective support, utilization, and total support. Scores on the SSRS range from 12 to 66, with higher scores indicating greater levels of social support. Specifically, scores of 22 or lower indicate low support, scores between 23 and 44 indicate moderate support, and scores of 45 or higher signify high social support (Fang et al., 2022).

The Mini Nutritional Assessment - Short Form (MNA-SF) (Rubenstein et al., 2001) was utilized to evaluate the nutritional status of elderly participants. Comprising 6 items, the MNA-SF scores range from 0 to 14, where 12–14 points indicate a normal nutritional state, and ≤ 11 points suggest malnutrition or the risk there of. The Barthel Index (BI) was utilized to assess participants’ basic activities of daily living (ADL). The BI evaluation comprises ten items: bowel control, grooming, toilet use, feeding, bathing, dressing, transferring, ambulation, and stair climbing. The scoring system is as follows: 100 points indicate full independence; 75–95 points reflect mild dependence; 50–70 points signify moderate dependence; 25–45 points represent severe dependence; and 0–20 points denote total dependence (Strini et al., 2020). Loneliness of elderly participants was assessed using a single - item question “Do you feel lonely?.” Participants had the option to select from five possible responses: never (0 points), rarely (1 point), sometimes (2 points), often (3 points), and always (4 points). The obtained scores were categorized as follows: 0–1 points indicating the absence of loneliness, and 2–4 points signifying the presence of loneliness (Bondevik and Skogstad, 1998; Drageset et al., 2011). The Short Physical Performance Battery (SPPB) (Corsonello et al., 2012), an objective measure developed by the U.S. National Institute on Aging, was administered. This test comprises 3 components: a balance assessment, a walking speed evaluation, and a chair stand test. Scores are scaled from 0 to 12, where 0–6 points denote poor physical function, 7–9 points suggest moderate function, and 10–12 points indicate good physical function. The Lubben Social Network Scale (LSNS) was applied to measure the social connectivity of the elderly, encompassing family, kinship, and friendships, as well as their overall social engagement (Lubben, 1988). A score of 19 or lower suggests a potential risk of social isolation, with lower scores reflecting a higher risk. The scale’s maximum score is 50, where scores below 20 denote a deficient social network, and scores of 20 or above signify an adequate social network. The Generalized Anxiety Disorder Scale (GAD-2), which is derived from the initial two items of the GAD-7, was employed due to its straightforward design and established reliability and validity in preliminary anxiety screening (Donker et al., 2011). Scores on the GAD-2 range from 0 to 6, with a score of 3 or higher indicating the presence of anxiety among the elderly. Additionally, the Tinetti Balance Scale, which evaluates both balance and gait, was utilized in this study (Opara et al., 2017). This scale is scored out of 28, with higher scores signifying superior mobility and balance; a score below 24 is indicative of a balance disorder.

Depressive symptoms were assessed using the Geriatric Depression Scale (GDS-15) (Zhang et al., 2020). The 15-item scale evaluates depressive symptoms and classifies participants into categories: scores of 0–4 indicate no depression, 5–8 suggest mild depression, 9–11 indicate moderate depression, and 12–15 signify severe depression. Participants were accordingly grouped based on their GDS-15 scores into depressed and non-depressed categories for the analysis. In this study, elderly with cognitive decline and GDS-15 scores ≥5 were included in the depression group (*n* = 128). Older adults with cognitive decline < 5 were included in the non-depressed group (*n* = 401).

### Sample size estimation

It is generally recommended that the sample size be at least ten times the number of variables to ensure robust statistical power (van Smeden et al., 2016). In this analysis, 15 independent variables were selected, which encompassed demographic characteristics, health status, and social determinants, including gender, age, education level, living arrangements, marital status, comorbidities, sleep quality, nutritional status, daily activities, loneliness, social support, physical health, social engagement, anxiety, and balance. Anticipating a 10% non-response rate, the sample size was calculated to include a minimum of 165 participants to ensure the accuracy and reliability of the study findings.

### Statistical analysis

Statistical analysis was performed using SPSS version 26.0 software (IBM Corp., Armonk, New York, USA). For continuous variables, normality tests were conducted, if the data follow a normal distribution, continuous variables are presented as mean ± SD and compared using Student’s *t*-test, while non-normally distributed variables are presented as median (inter-quartile range, IQR) and compared using Mann–Whitney U test. Categorical variables are expressed as numbers and percentages, and the chi-squared (*χ*^2^) test was used for comparison between groups. Multivariate logistic regression was employed to identify factors associated with depression in cognitively frail elderly individuals. Hierarchical regression analysis was used to explore the mediating role of sleep quality in the relationship between ADL and depression in the elderly with cognitive decline.

## Results

### Baseline information

540 elderly participants with cognitive frailty have been included ultimately in this study, based on screening 5,253 elderly adults from communities of Beijing in China. A total of 540 questionnaires were distributed, resulting in 529 valid responses, which corresponds to an effective recovery rate of 98.0%. Eleven questionnaires were classified as invalid: two due to incompleteness, three due to contradictions, and six due to unusually brief completion times. Among them, 128 participants (24.20%) were in the depressed group and 401 participants (75.80%) were in the non-depressed group (Table 1).

**Table 1:** Baseline characteristics of the study population.

Characteristics		Total	Depression	Non-Depression	*p*
Gender	Female	321 (60.7)	77 (60.2)	244 (60.8)	0.889
	Male	208 (39.3)	51 (39.8)	157 (39.2)	
Age	60–74	97 (0.2)	71 (17.7)	26 (20.3)	0.711
	75–80	110 (0.2)	81 (20.2)	29 (22.7)	
	81–85	173 (32.3)	136 (33.9)	37 (28.9)	
	>85	149 (57.5)	113 (28.2)	36 (28.1)	
Education	Illiterate/Primary	131 (24.8)	27 (21.1)	104 (25.9)	0.572
	Middle School	128 (24.2)	36 (28.1)	92 (22.9)	
	High School/Tech	88 (16.6)	21 (16.4)	67 (16.7)	
	College/Undergrad or above	182 (34.4)	44 (34.4)	138 (34.4)	
Marital status	Married/Remarried	354 (66.9)	85 (66.4)	269 (67.1)	0.887
	Single/Divorced/Widowed	175 (33.1)	43 (33.6)	132 (32.9)	
Living arrangement	Living along	75 (14.2)	19 (14.8)	56 (14.0)	0.804
	Not living alone	454 (85.8)	109 (85.2)	345 (86.0)	
Comorbidities	No	165 (31.2)	38 (29.7)	127 (31.7)	0.673
	Yes	364 (68.8)	90 (70.3)	274 (68.3)	
Nutritional status	Normal	354 (66.9)	73 (57.0)	281 (70.1)	0.006
	Malnutrition/risk	175 (33.1)	55 (43.0)	120 (29.9)	
ADL	Complete/severe dependency	29 (5.5)	22 (17.2)	7 (1.7)	<0.001
	Moderate dependency	130 (24.6)	95 (74.2)	35 (8.7)	
	Mild dependency	164 (31.0)	8 (6.3)	156 (38.9)	
	Independent	206 (38.9)	3 (2.3)	203 (50.6)	
Loneliness	No	290 (54.8)	16 (12.5)	274 (68.3)	<0.001
	Yes	239 (45.2)	112 (87.5)	127 (31.7)	
Social support	Low	74 (14.0)	11 (8.6)	63 (15.7)	0.043
	Medium/high	455 (86.0)	117 (91.4)	338 (84.2)	
Sleep quality	No disorder	237 (44.8)	16 (12.5)	221 (55.1)	<0.001
	Suspected insomnia	220 (41.6)	77 (60.2)	143 (35.7)	
	Insomnia	72(13.6)	35(27.3)	37(9.2)	
Physical condition	Good	175 (33.1)	38 (29.7)	137 (34.2)	0.127
	Moderate	109 (20.6)	21 (16.4)	88 (21.9)	
	Poor	245 (46.3)	69 (53.9)	176 (43.9)	
Social interaction	Normal	448 (84.7)	104 (81.3)	344 (85.8)	0.215
	At risk of isolation	81 (15.3)	24 (18.8)	57 (14.2)	
Anxiety	Yes	273 (51.6)	64 (50.0)	209 (52.1)	0.676
	No	256 (48.4)	64 (50.0)	192 (47.9)	
Balance and gait	Normal	199 (37.6)	43 (33.6)	156 (38.9)	0.280
	Balance disorder	330 (62.4)	85 (66.4)	245 (61.1)	

ADL, Activities of Daily Living.

### Risk factors

The multivariate logistic regression analysis revealed that ADL [Mild Dependence: OR = 176.729 (95% CI 32.427–963.172), *p* < 0.001; Moderate Dependence: OR = 51.769 (95% CI 12.541–213.697), *p* < 0.001], loneliness [OR = 13.821 (95% CI 6.095–31.338), *p* < 0.001], and sleep quality [Suspected Insomnia: OR = 7.310 (95% CI 2.316–23.074), *p* = 0.001] were significant risk factors for depression among cognitively frail elderly individuals (Table 2).

**Table 2:** Multivariate logistic regression analysis of factors influencing depression in elderly individuals with cognitive frailty.

Variables	*OR*	95% CI	*p-*value
Constant			0.009
ADL			
Independent	Ref.		
Mild dependence	176.729	32.427–963.172	<0.001
Moderate dependence	51.769	12.541–213.697	<0.001
Severe/complete dependence	0.886	0.259–3.032	0.847
Loneliness			
No	Ref.		
Yes	13.821	6.095–31.338	<0.001
Sleep quality			
No disorder	Ref.		
Suspected insomnia	7.310	2.316–23.074	0.001
Insomnia	1.694	0.615–4.665	0.308

ADL, Activities of Daily Living.

### Mediation analysis of sleep quality in the relationship between ADL and depression

**Figure 1:**
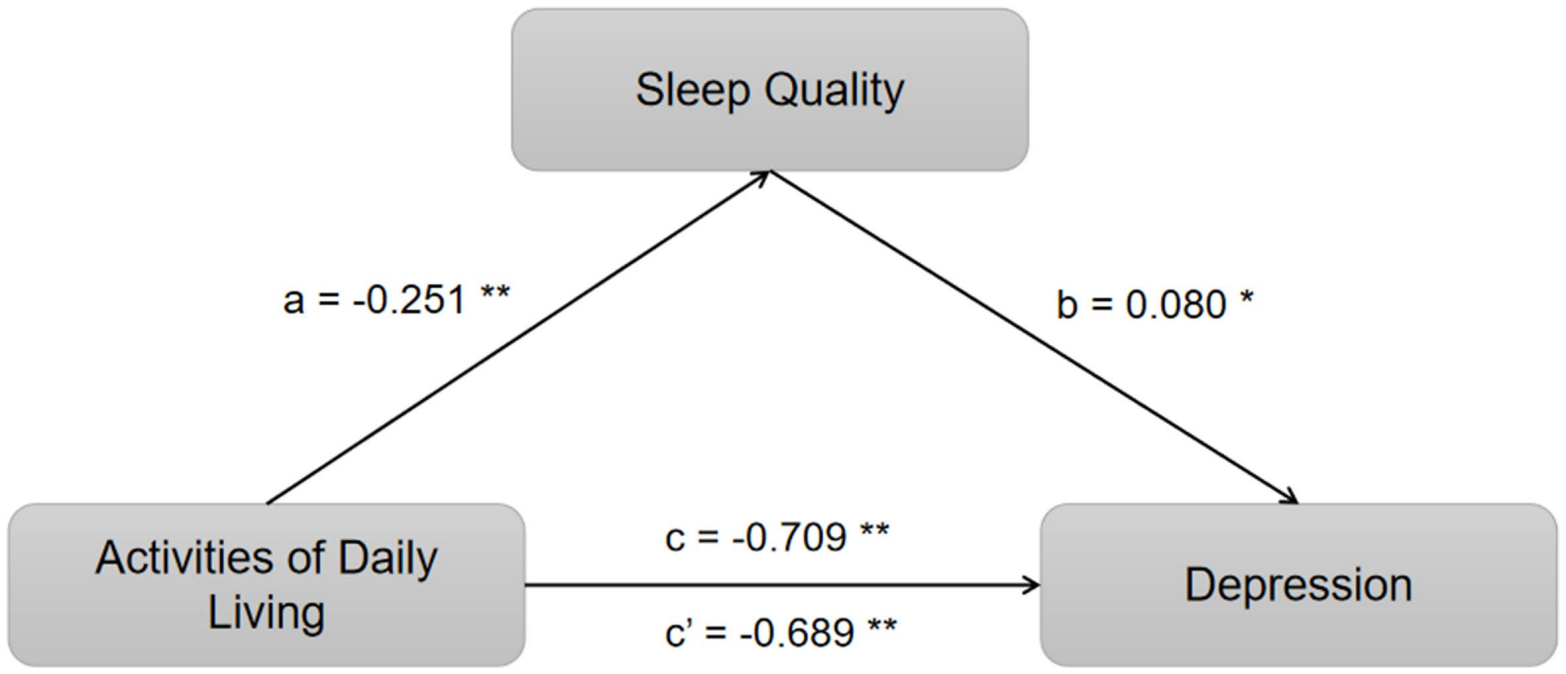
Mediation of sleep quality on the relationship between ADL and depression. *p < 0.05 and **p < 0.01.

Employing hierarchical regression for a stepwise analysis, this study identified significant associations: the impact of ADL on sleep quality (a = −0.251, *t* = −5.953, *p* < 0.05), the influence of ADL on depression (c = −0.709, *t* = −23.058, *p* < 0.05), the effect of sleep quality on depression (b = 0.080, *t* = 2.518, *p* < 0.05), and the direct effect of ADL on depression (c’ = −0.689, *t* = −21.800, *p* < 0.05). The mediation analysis is comprehensively detailed in Table 3, demonstrating that sleep quality partially mediates the relationship between ADL and depression. A summary of these findings is presented in Table 3 and visually represented in Figure 1.

**Table 3:** Analysis of the mediating effect of sleep quality on the relationship between ADL and depression.

Variables	Step one (dependent variable: sleep quality)		Step two (dependent variable: depression)		Step three (dependent variable: depression)	
	*β*	*t*	*β*	*t*	*β*	*t*
ADL	a = −0.251	−5.953**	c = −0.709	−23.058**	c’ = −0.689	−21.800**
Sleep Quality					b = 0.080	2.518*
*R*^2^	0.061		0.501		0.538	
*F*	35.443**		531.668**		271.698**	

*p < 0.05; **p < 0.01; ADL, Activities of Daily Living.

## Discussion

The results of this study indicate that ADL, sleep quality, and loneliness may have significant associations with depression in cognitively impaired older adults. Furthermore, the study revealed that sleep quality serves as a partial mediator in the relationship between ADL and depression. These associations underscore the potential benefit of interventions designed to enhance functional abilities, improve sleep quality, and mitigate social isolation in order to alleviate depressive symptoms among older adults with cognitive frailty (see Table 4).

**Table 4:** Mediation effect testing of sleep quality on the relationship between ADL and depression.

Independent variables	Sleep quality model 1	Depression	
		Model 2	Model 3
ADL	a = −0.251**	c = −0.709**	c’ = −0.689**
Sleep Quality	—	—	b = 0.080*
*R*^2^	0.061	0.501	0.538
*F*	35.443**	531.668**	271.698**

*p < 0.05; **p < 0.01; ADL, Activities of Daily Living.

Multivariate logistic regression analysis from this study confirms that a higher score on ADL serves as a protective factor against depression in elderly individuals with cognitive frailty, aligning with the research by Johansson et al. (2021), which underscores the correlation between diminished self-care capabilities and an elevated risk of depression. A longitudinal study from Singapore reported a twelvefold increase in Basic ADL disability and a twofold increase in Instrumental ADL disability among those with cognitive frailty (Feng et al., 2017), highlighting the rapid decline in functional capacity associated with cognitive decline. The decline in physiological function due to the degradation of organ and tissue structures in cognitively frail seniors can precipitate a loss of self-care ability, potentially leading to feelings of helplessness and frustration. Such emotional states may trigger or intensify depression, thereby exacerbating cognitive frailty. This underscores the importance of early interventions that focus on maintaining or improving ADL capabilities in older adults with cognitive frailty (Gao et al., 2021). By doing so, we may not only mitigate the risk of depression but also slow the progression of cognitive decline. Furthermore, understanding the interplay between ADL, depression, and cognitive frailty offers valuable insights for developing comprehensive care plans that address the multifaceted needs of this vulnerable population.

The relationship between sleep quality and depressive symptoms in elderly individuals with cognitive frailty is complex and multifaceted. Firstly, sleep disturbances have been identified as a significant risk factor for depression in older adults with cognitive frailty, which aligns with Bao et al.’s (2017) findings that highlight the potential for sleep disorders to trigger a cascade of cardiovascular, physical, and mental health issues. Moreover, the interplay between sleep quality, ADL, and depression is underscored by the research indicating that sleep problems can contribute to conditions like hypertension, diabetes, and obesity, as well as restrict ADL, consequently impacting the health-related quality of life (HRQOL) of older adults (Ge et al., 2019; Zendels et al., 2021). This is particularly relevant as sleep issues are common among older adults, with estimates suggesting that more than 40% face inadequate sleep quality, which can further exacerbate depressive symptoms (Leng et al., 2024). Therefore, improving sleep quality emerges as a potentially effective strategy for the prevention and treatment of depression within this specific population.

Loneliness is a notable risk factor for depression among older adults with cognitive frailty, potentially mediating this relationship by undermining social support systems and intensifying psychological distress, as supported by Wang et al.’s (2024) research. Wang’s findings underscore the severe impact of social isolation on the mental health of this demographic. The presence of a nurturing family environment and robust intergenerational care is crucial for mitigating negative emotional states and psychological strain, which can contribute to delaying cognitive decline (Edelman et al., 2012). A recent study suggests that the impact of social support on cognitive frailty may be exerted through its effects on depressive symptoms (Ma et al., 2022). However, the present study did not observe a significant influence of social support on depression in cognitively frail elderly individuals. Further research is warranted to explore the multifaceted aspects of social support and their complex interplay with depression in the context of cognitive frailty, aiming to reveal more sophisticated insights into this dynamic.

The mediation analysis from this study reveals that sleep quality partially mediates the impact of ADL on depressive symptoms in elderly individuals with cognitive frailty. ADL directly predict depressive symptoms and also directly influence sleep quality. This relationship is further nuanced by the mediating role of sleep quality in the association between ADL and psychological distress, as highlighted in the study by Zhang et al. (2022), which found that ADL and depression exhibited a chain mediation effect on this relationship. Moreover, physical activity was identified as a factor that significantly reduces the mediating influence of ADL on the relationship between sleep quality and psychological distress, suggesting that maintaining an active lifestyle may be a crucial component in managing depressive symptoms in older adults with cognitive frailty (Zhang et al., 2022). These findings underscore the complexity of the interactions between sleep, ADL, and depression, and emphasize the importance of considering these factors holistically when developing interventions for older adults. Even when accounting for sleep quality as a mediator, ADL retains a significant predictive relationship with depressive symptoms, and sleep quality itself significantly predicts depressive symptoms. This relationship is attributed to two primary factors: First, the general decline in physical function in older adults often results in poor health and heightened disability risk. As self-care abilities diminish, increased dependency on others for both personal care and external activities can lead to significant lifestyle changes, creating emotional distress that may trigger or intensify depressive symptoms (Liu et al., 2023; Zou C. et al., 2023; Zou Z. et al., 2023). Second, sleep disorders compromise sleep quality and metabolism, potentially aggravating neurological dysfunction and hormonal imbalances, which can exacerbate depression. The interplay between depression and sleep is complex and bidirectional, with insomnia particularly worsening depressive symptoms (Liu et al., 2022). However, the precise mechanisms underlying these relationships warrant further investigation. This complex interplay underscores the need for a comprehensive understanding of the factors that interconnect sleep quality, ADL, and depression in the context of cognitive frailty, particularly in older adults.

Depression in older adults with cognitive frailty is indeed a significant concern that warrants attention. Global research indicates that the prevalence of depression is 1.8% among self-sufficient seniors, escalating to 7.2% in those aged 75 and above (Luppa et al., 2012). Our study observed a higher proportion of depressive symptoms, with 24.20% of elderly individuals with cognitive frailty affected. The majority of these cases (93.33%) were characterized as mild, while a smaller proportion (6.67%) were moderate. This observation underscores the need for vigilant monitoring. The interplay between cognitive decline and decreased physical activity can foster negative emotional states, including depression, which can further aggravate physical inactivity, diminish social participation, and contribute to malnutrition, thereby escalating the risk of cognitive frailty (Choi et al., 2019; Liu et al., 2022). The concurrent presence of frailty and depression poses a risk for severe cognitive decline, increased disability, and mortality. It is recommended that healthcare professionals strengthen the assessment of cognitive frailty and depression in the elderly and engage in comprehensive health management strategies, such as ongoing mental health assessments and educational interventions, to alleviate depressive symptoms and improve the quality of life for this vulnerable demographic.

The limitations of this study should be acknowledged. Firstly, due to constraints on time and resources, the study utilized a convenience sample of elderly participants from six communities in Beijing, which may introduce selection bias. Future research should employ multi-stage stratified random sampling to enhance data diversity. Secondly, the cross-sectional design limits the ability to establish causality between self-care ability, self-assessed health, and depressive symptoms; longitudinal cohort studies are suggested for more definitive conclusions. Thirdly, while the study explores the mediating role of sleep quality between ADL and depression, the actual mechanisms are likely more complex. Future investigations should consider additional potential mediators in the relationship between self-care ability and depressive symptoms among the elderly.

In conclusion, this study conducted a comprehensive analysis of the factors associated with depression in elderly individuals with cognitive frailty. It identified key risk factors such as ADL, loneliness, and sleep quality, and revealed a mediating effect of sleep quality on the relationship between ADL and depression. The findings suggest that enhancing ADL, improving sleep quality, and reducing loneliness could significantly decrease the risk of depression in this population. These insights are essential for creating predictive models and tailored interventions addressing cognitive frailty in older adults.

## Data Availability

The raw data supporting the conclusions of this article will be made available by the authors, without undue reservation.

## References

[ref1] Abizanda P. Romero L. Sánchez-Jurado P. M. Ruano T. F. Ríos S. S. Sánchez M. F. (2016). Energetics of aging and frailty: the FRADEA study. J. Gerontol. A Biol. Sci. Med. Sci. 71, 787–796. doi: 10.1093/gerona/glv182, PMID: 26463762 PMC4888388

[ref2] Alkhodary A. A. Aljunid S. M. Ismail A. Nur A. M. Shahar S. (2022). Health care utilization and out-of-pocket payments among elderly with cognitive frailty in Malaysia. Int. J. Environ. Res. Public Health 19:3361. doi: 10.3390/ijerph19063361, PMID: 35329059 PMC8954898

[ref3] Bao Y. P. Han Y. Ma J. Wang R. J. Shi L. Wang T. Y.. (2017). Cooccurrence and bidirectional prediction of sleep disturbances and depression in older adults: Meta-analysis and systematic review. Neurosci. Biobehav. Rev. 75, 257–273. doi: 10.1016/j.neubiorev.2017.01.032, PMID: 28179129

[ref4] Boga S. M. Saltan A. (2020). Identifying the relationship among sleep, mental status, daily living activities, depression and pain in older adults: a comparative study in Yalova. Turkey. J. Pak. Med. Assoc. 70, 236–242. doi: 10.5455/JPMA.301384, PMID: 32063613

[ref5] Bondevik M. Skogstad A. (1998). The oldest old, ADL, social network, and loneliness. West. J. Nurs. Res. 20, 325–343. doi: 10.1177/019394599802000305, PMID: 9615601

[ref6] Choi K. W. Chen C. Y. Stein M. B. Klimentidis Y. C. Wang M. J. Koenen K. C.. (2019). Assessment of bidirectional relationships between physical activity and depression among adults: a 2-sample Mendelian randomization study. JAMA Psychiatry 76, 399–408. doi: 10.1001/jamapsychiatry.2018.4175, PMID: 30673066 PMC6450288

[ref7] Corsonello A. Lattanzio F. Pedone C. Garasto S. Laino I. Bustacchini S.. (2012). Prognostic significance of the short physical performance battery in older patients discharged from acute care hospitals. Rejuvenation Res. 15, 41–48. doi: 10.1089/rej.2011.1215, PMID: 22004280 PMC3283437

[ref8] Donker T. van Straten A. Marks I. Cuijpers P. (2011). Quick and easy self-rating of generalized anxiety disorder: validity of the Dutch web-based GAD-7, GAD-2 and GAD-SI. Psychiatry Res. 188, 58–64. doi: 10.1016/j.psychres.2011.01.016, PMID: 21339006

[ref9] Drageset J. Kirkevold M. Espehaug B. (2011). Loneliness and social support among nursing home residents without cognitive impairment: a questionnaire survey. Int. J. Nurs. Stud. 48, 611–619. doi: 10.1016/j.ijnurstu.2010.09.008, PMID: 20947083

[ref10] Edelman S. Mahoney A. E. Cremer P. D. (2012). Cognitive behavior therapy for chronic subjective dizziness: a randomized, controlled trial. Am. J. Otolaryngol. 33, 395–401. doi: 10.1016/j.amjoto.2011.10.009, PMID: 22104568

[ref11] Fang J. Ren J. Ren L. Qiu X. Yuan S. Wang W.. (2022). Perceived social support and associated factors among community-dwelling older adults with frailty and pre-frailty in Hangzhou, China. Front. Psych. 13:944293. doi: 10.3389/fpsyt.2022.944293, PMID: 35911254 PMC9329702

[ref12] Feng L. Zin Nyunt M. S. Gao Q. Feng L. Yap K. B. Ng T. P. (2017). Cognitive frailty and adverse health outcomes: findings from the Singapore longitudinal ageing studies (SLAS). J. Am. Med. Dir. Assoc. 18, 252–258. doi: 10.1016/j.jamda.2016.09.015, PMID: 27838339

[ref13] Gao Q. Hu K. Yan C. Zhao B. Mei F. Chen F.. (2021). Associated factors of sarcopenia in community-dwelling older adults: a systematic review and Meta-analysis. Nutrients 13:4291. doi: 10.3390/nu13124291, PMID: 34959843 PMC8707132

[ref14] Ge Y. Xin S. Luan D. Zou Z. Liu M. Bai X.. (2019). Association of physical activity, sedentary time, and sleep duration on the health-related quality of life of college students in Northeast China. Health Qual. Life Outcomes 17:124. doi: 10.1186/s12955-019-1194-x, PMID: 31311564 PMC6636029

[ref15] Johansson P. Jaarsma T. Andersson G. Lundgren J. (2021). The impact of internet-based cognitive behavioral therapy and depressive symptoms on self-care behavior in patients with heart failure: a secondary analysis of a randomised controlled trial. Int. J. Nurs. Stud. 116:103454. doi: 10.1016/j.ijnurstu.2019.103454, PMID: 31727306

[ref16] Karam C. (2022). Pain, sleep, fatigue, and activities of daily living in patients with neuropathy. Muscle Nerve 66, 380–381. doi: 10.1002/mus.27692, PMID: 35919957

[ref17] Kendzerska T. B. Smith P. M. Brignardello-Petersen R. Leung R. S. Tomlinson G. A. (2014). Evaluation of the measurement properties of the Epworth sleepiness scale: a systematic review. Sleep Med. Rev. 18, 321–331. doi: 10.1016/j.smrv.2013.08.002, PMID: 24135493

[ref18] Leng Y. Knutson K. Carnethon M. R. Yaffe K. (2024). Association between sleep quantity and quality in early adulthood with cognitive function in midlife. Neurology 102:e208056. doi: 10.1212/WNL.0000000000208056, PMID: 38170947 PMC10870739

[ref19] Liu H. Ma Y. Lin L. Sun Z. Li Z. Jiang X. (2023). Association between activities of daily living and depressive symptoms among older adults in China: evidence from the CHARLS. Front. Public Health 11:1249208. doi: 10.3389/fpubh.2023.1249208, PMID: 38035294 PMC10687586

[ref20] Liu X. Xia X. Hu F. Hao Q. Hou L. Sun X.. (2022). The mediation role of sleep quality in the relationship between cognitive decline and depression. BMC Geriatr. 22:178. doi: 10.1186/s12877-022-02855-5, PMID: 35236297 PMC8890949

[ref21] Lubben J. E. (1988). Assessing social networks among elderly populations. Fam. Community Health 11, 42–52. doi: 10.1097/00003727-198811000-00008

[ref22] Luppa M. Sikorski C. Luck T. Ehreke L. Konnopka A. Wiese B.. (2012). Age-and gender-specific prevalence of depression in latest-life--systematic review and meta-analysis. J. Affect. Disord. 136, 212–221. doi: 10.1016/j.jad.2010.11.033 21194754

[ref23] Ma W. Wu B. Gao X. Zhong R. (2022). Association between frailty and cognitive function in older Chinese people: a moderated mediation of social relationships and depressive symptoms. J. Affect. Disord. 316, 223–232. doi: 10.1016/j.jad.2022.08.032, PMID: 35988782

[ref24] Martin F. C. O'Halloran A. M. (2020). Tools for assessing frailty in older people: general concepts. Adv. Exp. Med. Biol. 1216, 9–19. doi: 10.1007/978-3-030-33330-0_2, PMID: 31894542

[ref25] Nasreddine Z. S. Phillips N. A. Bédirian V. Charbonneau S. Whitehead V. Collin I.. (2005). The Montreal cognitive assessment, MoCA: a brief screening tool for mild cognitive impairment. J. Am. Geriatr. Soc. 53, 695–699. doi: 10.1111/j.1532-5415.2005.53221.x, PMID: 15817019

[ref26] Opara J. Małecki A. Małecka E. Socha T. (2017). Motor assessment in Parkinson’s disease. Ann. Agric. Environ. Med. 24, 411–415. doi: 10.5604/12321966.1232774 28954481

[ref27] Panza F. Lozupone M. Solfrizzi V. Sardone R. Dibello V. Di Lena L.. (2018). Different cognitive frailty models and health- and cognitive-related outcomes in older age: from epidemiology to prevention. J. Alzheimers Dis. 62, 993–1012. doi: 10.3233/JAD-170963, PMID: 29562543 PMC5870024

[ref28] Qiu Y. Li G. Wang X. Zheng L. Wang C. Wang C.. (2022). Prevalence of cognitive frailty among community-dwelling older adults: a systematic review and meta-analysis. Int. J. Nurs. Stud. 125:104112. doi: 10.1016/j.ijnurstu.2021.104112, PMID: 34758429

[ref29] Ren X. Q. Zhao G. M. Fang S. W. Xu L. F. Wang L. D. Zhao L. H.. (2024). Mediating roles of activities of daily living and depression on the relationship between sleep quality and health-related quality of life. Sci. Rep. 14:14057. doi: 10.1038/s41598-024-65095-0, PMID: 38890451 PMC11189409

[ref30] Rivan N. F. M. Singh D. K. A. Shahar S. Wen G. J. Rajab N. F. Din N. C.. (2021). Cognitive frailty is a robust predictor of falls, injuries, and disability among community-dwelling older adults. BMC Geriatr. 21:593. doi: 10.1186/s12877-021-02525-y, PMID: 34696720 PMC8543922

[ref31] Rubenstein L. Z. Harker J. O. Salvà A. Guigoz Y. Vellas B. (2001). Screening for undernutrition in geriatric practice: developing the short-form mini-nutritional assessment (MNA-SF). J. Gerontol. A Biol. Sci. Med. Sci. 56, M366–M372. doi: 10.1093/gerona/56.6.m366, PMID: 11382797

[ref32] Soldatos C. R. Dikeos D. G. Paparrigopoulos T. J. (2020). Athens insomnia scale:validation of an instrument based on ICD-10 criteria. J. Psychosom. Res. 48, 555–560. doi: 10.1016/s0022-3999(00)00095-7, PMID: 11033374

[ref33] Strini V. Piazzetta N. Gallo A. Schiavolin R. (2020). Barthel index: creation and validation of two cut-offs using the BRASS index. Acta Biomed 91, 19–26. doi: 10.23750/abm.v91i2-S.9226, PMID: 32168309 PMC7944663

[ref34] Sugimoto T. Arai H. Sakurai T. (2022). An update on cognitive frailty: its definition, impact, associated factors and underlying mechanisms, and interventions. Geriatr Gerontol Int 22, 99–109. doi: 10.1111/ggi.14322, PMID: 34882939

[ref35] Taylor H. O. Taylor R. J. Nguyen A. W. Chatters L. (2018). Social isolation, depression, and psychological distress among older adults. J. Aging Health 30, 229–246. doi: 10.1177/0898264316673511, PMID: 28553785 PMC5449253

[ref36] van Smeden M. de Groot J. A. Moons K. G. Collins G. S. Altman D. G. Eijkemans M. J.. (2016). No rationale for 1 variable per 10 events criterion for binary logistic regression analysis. BMC Med. Res. Methodol. 16:163. doi: 10.1186/s12874-016-0267-3, PMID: 27881078 PMC5122171

[ref37] Wang S. Lin J. Kuang L. Yang X. Yu B. Cui Y. (2024). Risk factors for social isolation in older adults: a systematic review and meta-analysis. Public Health Nurs. 41, 200–208. doi: 10.1111/phn.13266, PMID: 38037451

[ref38] Wu Y. Li S. Han D. Zhang M. Zhao J. Liao H.. (2023). The mediating role of depression in association between Total sleep time and instrumental activities of daily living in China. Int. J. Public Health 68:1605678. doi: 10.3389/ijph.2023.1605678, PMID: 37081904 PMC10110912

[ref39] Zacková L. Jáni M. Brázdil M. Nikolova Y. S. Marečková K. (2021). Cognitive impairment and depression: meta-analysis of structural magnetic resonance imaging studies. Neuroimage Clin. 32:102830. doi: 10.1016/j.nicl.2021.102830, PMID: 34560530 PMC8473769

[ref40] Zendels P. Moore-Harrison T. Gaultney J. F. (2021). Sleep and risk for metabolic syndrome, hypertension, diabetes and obesity among community-dwelling older adults. Int. J. Exerc. Sci. 15, 88–102. doi: 10.70252/ODDB8638, PMID: 36895436 PMC9987437

[ref41] Zhang C. Xiao S. Lin H. Shi L. Zheng X. Xue Y.. (2022). The association between sleep quality and psychological distress among older Chinese adults: a moderated mediation model. BMC Geriatr. 22:35. doi: 10.1186/s12877-021-02711-y, PMID: 35012479 PMC8744230

[ref42] Zhang C. Zhang H. Zhao M. Liu D. Zhao Y. Yao Y. (2020). Assessment of geriatric depression Scale's applicability in Longevous persons based on classical test and item response theory. J. Affect. Disord. 274, 610–616. doi: 10.1016/j.jad.2020.05.090, PMID: 32663994

[ref43] Zhang Y. Zhou J. J. Zhang X. M. Liu J. T. Li M. R. Liang J. Y.. (2023). Management of cognitive frailty: a network meta-analysis of randomized controlled trials. Int. J. Geriatr. Psychiatry 38:e5994. doi: 10.1002/gps.5994, PMID: 37655500

[ref44] Zou Z. Wang Z. Herold F. Kramer A. F. Ng J. L. Hossain M. M.. (2023). Validity and reliability of the physical activity and social support scale among Chinese established adults. Complement. Ther. Clin. Pract. 53:101793. doi: 10.1016/j.ctcp.2023.101793, PMID: 37579659

[ref45] Zou C. Yu Q. Wang C. Ding M. Chen L. (2023). Association of depression with cognitive frailty: a systematic review and meta-analysis. J. Affect. Disord. 320, 133–139. doi: 10.1016/j.jad.2022.09.118, PMID: 36183817

